# Visfatin: A Possible Role in Cardiovasculo-Metabolic Disorders

**DOI:** 10.3390/cells9112444

**Published:** 2020-11-09

**Authors:** Ali Dakroub, Suzanne A. Nasser, Nour Younis, Humna Bhagani, Yusra Al-Dhaheri, Gianfranco Pintus, Assaad A. Eid, Ahmed F. El-Yazbi, Ali H. Eid

**Affiliations:** 1 Department of Pharmacology and Toxicology, Faculty of Medicine, American University of Beirut, Beirut P.O. Box 11-0236, Lebanon; ahd31@mail.aub.edu (A.D.); nky02@mail.aub.edu (N.Y.); hxb04@mail.aub.edu (H.B.); ae88@aub.edu.lb (A.F.E.-Y.); 2 Department of Pharmacology and Therapeutics, Beirut Arab University, Beirut P.O. Box 11-5020, Lebanon; san413@bau.edu.lb; 3 Department of Biology, College of Science, United Arab Emirates University, Al-Ain P.O. Box 15551, UAE; yusra.aldhaheri@uaeu.ac.ae; 4 Department of Medical Laboratory Sciences, University of Sharjah, Sharjah P.O. Box 27272, UAE; gpintus@sharjah.ac.ae; 5 Department of Biomedical Sciences, University of Sassari, Viale San Pietro 43, 07100 Sassari, Italy; 6 Department of Anatomy, Cell Biology and Physiological Sciences, Faculty of Medicine, American University of Beirut, Beirut P.O. Box 11-0236, Lebanon; ae49@aub.edu.lb; 7 Department of Pharmacology and Toxicology, Alexandria University, Alexandria 21521, El-Mesallah, Egypt; 8 Department of Basic Medical Sciences, College of Medicine, QU Health, Qatar University, Doha P.O. Box 2713, Qatar; 9 Biomedical and Pharmaceutical Research Unit, QU Health, Qatar University, Doha P.O. Box 2713, Qatar

**Keywords:** visfatin/NAMPT, metabolic disorders, NAD^+^, sirtuins, PARPs/MARTs

## Abstract

Visfatin/NAMPT (nicotinamide phosphoribosyltransferase) is an adipocytokine with several intriguing properties. It was first identified as pre-B-cell colony-enhancing factor but turned out to possess enzymatic functions in nicotinamide adenine dinucleotide biosynthesis, with ubiquitous expression in skeletal muscles, liver, cardiomyocytes, and brain cells. Visfatin exists in an intracellular (iNAMPT) and extracellular (eNAMPT) form. Intracellularly, visfatin/iNAMPT plays a regulatory role in NAD^+^ biosynthesis and thereby affects many NAD-dependent proteins such as sirtuins, PARPs, MARTs and CD38/157. Extracellularly, visfatin is associated with many hormone-like signaling pathways and activates some intracellular signaling cascades. Importantly, eNAMPT has been associated with several metabolic disorders including obesity and type 1 and 2 diabetes. In this review, a brief overview about visfatin is presented with special emphasis on its relevance to metabolic diseases. Visfatin/NAMPT appears to be a unique molecule with clinical significance with a prospective promising diagnostic, prognostic, and therapeutic applications in many cardiovasculo-metabolic disorders.

## 1. Introduction

The role of adipose tissues evolved from simply being the main reservoir of energy in the form of triglycerides to becoming an endocrine gland and essentially a part of the endocrine system. This is due to that fact that adipose tissues secrete hormone-like substances known as “adipokines” or “adipocytokine” [1]. Adipokines include inflammatory mediators such as complement factors B, C3, and D, haptoglobin, hepatocyte growth factor, adiponectin, prostaglandin E2, interleukin (IL)-1β, IL-6, IL-8, IL-10, leukemia inhibitory factor, macrophage migration inhibitory factor, tumor necrosis factor (TNF) and many more [2]. The concentration of these adipokines may be altered or dysregulated in some metabolic disorders, such as obesity [3,4] and type 2 diabetes [4], sepsis [5], cardiovascular disorders, such as hypertension and atherosclerosis [6,7,8,9] and many other cardiovasculo-metabolic disorders. The relation between metabolism and cardiovascular disease starts in utero and continues until adulthood [10].

**Figure 1:**
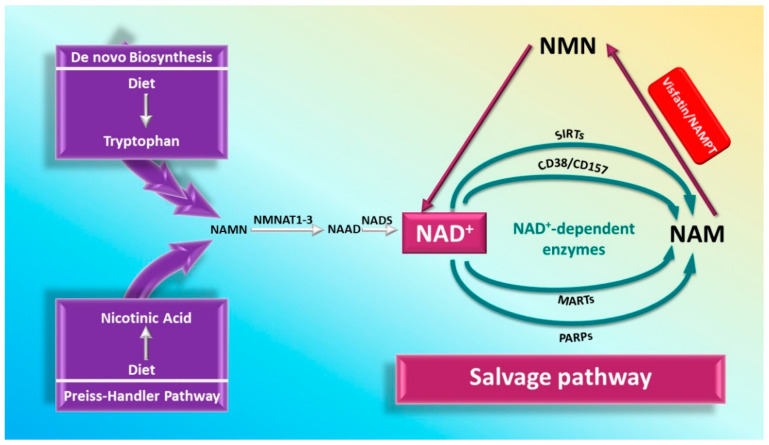
The three-mammalian nicotinamide adenine dinucleotide (NAD^+^) biosynthesis pathways. De novo synthesis and the Preiss–Handler pathway start from nutritionally-derived tryptophan (essential amino acid) and nicotinic acid (NA), respectively. Both will eventually yield nicotinic acid mononucleotide (NAMN), which will be converted to nicotinic acid adenine dinucleotide (NAAD) via nicotinamide mononucleotide adenylyltransferases (NMNATs). NAAD conversion to NAD^+^ is catalyzed by nicotinamide adenine dinucleotide synthetase (NADS). Nicotinamide (NAM) constitute an important precursor for NAD^+^ inside the cell via the salvage (rescue) pathway. NAM is the product of several NAD^+^ dependent enzymes: sirtuins (SIRTs), poly (ADP-ribose) polymerases (PARPs), mono (ADP-ribose) transferases (MARTs), cluster of differentiation 38 (CD38) and CD157. NAM will be converted to nicotinamide mononucleotide (NMN) in the rate determining step of the salvage pathway via nicotinamide phosphoribosyltransferase (NAMPT) or visfatin. NAD^+^ may be regenerated from NMN via nicotinamide mononucleotide adenylyltransferase (NMNAT).

In 1994, a protein, with a novel cytokine-like activity, was initially uncovered from the bone marrow cDNA library [11]. It was denoted the name, pre-B-cell colony-enhancing factor (PBEF), due to its enhancing role in murine pre-B-cell colony formation from early B lineage precursor cells [11]. In 2001, a gene, with a similar sequence to PBEF, known as nadV, was discovered to permit NAD-independent growth of Gram-negative bacteria such as *Haemophilus influenza* and *Actinobacillus* [12]. This shed light on a possible underlying role of PBEF in nicotinamide adenine dinucleotide (NAD) biosynthesis. In 2002, PBEF was identified to be a protein with enzymatic properties capable of catalyzing the synthesis of nicotinamide mononucleotide (NMN), an intermediate of NAD biosynthesis, from nicotinamide (NAM) and 5-phosphoribosyl-1-pyrophosphate (Figure 1) [13]. As a consequence, PBEF was renamed nicotinamide phosphoribosyltransferase (NAMPT). NAMPT is a dimeric type 2 phosphoribosyltransferase and its role in NAD biosynthesis has been emphasized [14]. In 2005, a study reported NAMPT or PBEF as being a protein that is secreted solely by visceral fat, hence it was denoted visfatin meaning visceral fat-specific adipokine [15]. The terms visfatin, PBEF, and NAMPT are nowadays used interchangeably.

## 2. Visfatin’s Tissue Expression

In 1994, cloning techniques revealed widespread expression and secretion of visfatin, in bone marrow, liver, muscles, heart, placenta, lung, and kidney tissues [11]. The liver and muscles expressed the highest amounts, with the former being the highest among all [11]. Visfatin/NAMPT was found to have ubiquitous expression in adipose tissues, liver, muscle, and immune cells [15,16,17]. Moreover, other studies reported that visfatin is expressed in myocardial cells, particularly in cardiomyocytes and cardiac fibroblasts in a similar fashion at the mRNA and protein levels [18]. In addition, visfatin is found in brain neuronal cells with marked up-regulation of expression during brain damage (ischemia) [19,20]. Hence, visfatin production is no longer considered limited to visceral fat and its ubiquitous expression and secretion in many other tissues suggests a vital role in their physiological processes.

The fact that visfatin possesses both cytokine-like extrinsic activity (PBEF) and an enzymatic intrinsic activity (NAMPT) incited researchers to investigate a possible determinant role in physiology and pathophysiology of cardiovasculo-metabolic disorders [21]. Moreover, the comparison of the visfatin gene in pigs and seven other representative organisms revealed that the visfatin gene is highly conserved among different organisms [17]. Several variants of visfatin have been reported. Some of the variants are present in all cells whereas others a more localized and absent in liver and testes [17]. This may be suggestive of specific functions performed by each variant.

## 3. Functional Roles

Visfatin/NAMPT can exist as intracellular (iNAMPT) or extracellular (eNAMPT) exerting different roles [16,22].

### 3.1. Intracellular Visfatin (iNAMPT)

Nicotinamide adenine dinucleotides are pyridine substrates that include NAD^+^ and NADP (phosphorylated form of NAD) and their reduced forms NADH and NADPH, respectively. They are small molecule co-factors, essential in energy metabolism: NAD and NADH in oxidative energy-releasing processes (catabolic reactions) and NADPH and NADP in reductive biosynthesis (anabolic reactions), detoxification and anti-oxidation [23,24,25,26,27]. NAD substrates serve as cofactors for several enzymes, which are known as NAD-dependent enzymes. These enzymes bridge the interplay between cellular metabolic processes and different epigenetic regulation mechanisms. This distinguishes NAD as a core potent substrate with tremendous regulatory and physiological functions metabolism [23,24,25,26,27]. NAD^+^ and NADP remain as essential cofactors to several cellular and metabolic processes pertinent to carbohydrates, proteins, lipid, cholesterol and steroids metabolism [24,25,27]. NAD^+^ is more dedicated to the breakdown (oxidation) of carbohydrates, fats, and proteins, and other reducing agents such as alcohols [24,25,27]. NADPH is primarily utilized in synthetic pathways involving fatty acids and cholesterol [24,25,26,27]. Functional roles o iNAMPT are discussed below and summarized in Table 1.

#### 3.1.1. Role of iNAMPT in NAD Biosynthesis

NAD biosynthesis has been shown to be a key player in basic cellular function [23,24,25,26,27]. There are three main pathways of NAD biosynthesis: (1) the de novo synthesis: starting from tryptophan (kinurenine pathway), (2) the salvage (rescue) pathway: synthesis from NAM, (3) and the Preiss–Handler pathway which involves generation from nicotinic acid (NA) [22,28,29] (Figure 1). The intracellular form of visfatin, iNAMPT, is involved in the salvage pathway [22,28,29]. The salvage pathway involves the transfer of a phosphoribosyl moiety from 5-phosphoribosyl-1-pyrophosphate to NAM (or possibly to nicotinamide riboside: NR) to yield nicotinamide mononucleotide (NMN). This reaction is catalyzed by Nicotinamide phosphoribosyltransferase (NAMPT) which turns to be a form of visfatin [22,28,29]. Then NMN may be converted to the ultimate NAD by the enzyme nicotinamide mononucleotide adenylyltransferase (NMNAT) which exists in several isoforms and requires ATP [22]. Interestingly NAMPT is the rate-limiting step in the NAD salvage pathway [21,30]. Unfortunately, the metabolic conditions and cellular processes that dictate the utilization or recruitment of the NAD salvage pathway remain unclear.

Cellular and metabolic processes require continuous activity of NAD-consuming enzymes. This prompts eukaryotic cells to resynthesize NAD from nictotinamide (NAM) using a salvage pathway. It should be noted that dietary intake of tryptophan or low amounts of niacin (less than 20 mg) is sufficient to fulfill the baseline needs for proper NAD biosynthesis [28]. The NAMPT-dependent salvage pathway remains a predominant pathway for NAD^+^ synthesis in mammals [31]. Notably, there is increasing evidence that increasing rates of NAD^+^ biosynthesis might have diverse protective roles against aging and stress [32,33], implicating a potential role of visfatin in regulating these processes.

#### 3.1.2. Visfatin and NAD-Dependent Enzymes

The ability of visfatin to regulate NAD^+^ synthesis makes it a dominant regulator of several cellular components, including sirtuins (SIRTs), poly (ADP-ribose) polymerase (PARPs), (CD38), and CD157 [22,34].

##### Sirtuins

**Figure 2:**
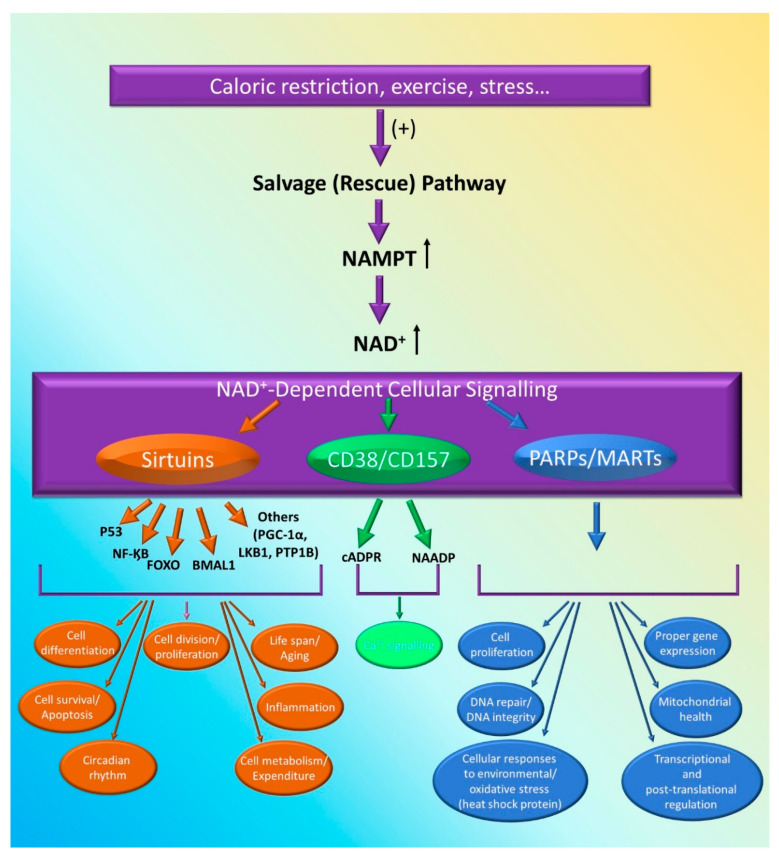
The salvage (rescue) pathway remains a predominant pathway to meet NAD+ cellular requirements. Several NAD^+^ dependent cellular signaling pathways exist and involve sirtuins, CD38/CD157, and PARPs/MARTs. They are essential for various cellular biological functions such as cellular division, proliferation, inflammation, maintaining genome integrity, DNA and protein synthesis, cellular anti-oxidative power, cellular metabolism, energy expenditure, mitochondrial health, and aging. FOXO: fork-head box class O; BMAL1: brain and muscle aryl hydrocarbon receptor nuclear translocator-like.

SIRTs are a group of enzymes that possess NAD-dependent protein deacetylase activity. Sirtuins have intrigued researchers due to their ability to regulate major metabolic processes and interfere with the lifespan [35]. Sirtuins constitute a family of seven proteins SIRT 1–7 [35,36] with different localization and activity within the cell. SIRT 1 is present in both the nucleus and cytosol, whereas SIRT2 is limited to cytosol and SIRT 6 to the nucleus exclusively [35,36]. Sirtuins 3–5 (SIRT 3–5) are present in the mitochondria and SIRT 7 in the nucleolus [35,36]. SIRTs perform different activities such as deacetylation (SIRT 1–3) and ADP-ribosylation (SIRT 4, 6). SIRTs also participate in mediating metabolic processes, such as glucose and lipids metabolism [35]. Additionally, SIRTs have wide ranging effects and associations with several processes such as apoptosis, inflammation, energy expenditure, insulin sensitivity and many other processes [37,38,39]. It seems that SIRT 1 induces some of its effects through interaction with transcription factors, such as fork-head box class O (FOXO), brain and muscle aryl hydrocarbon receptor nuclear translocator-like (BMAL1) [39], nuclear factor kappa B (NF-κB), and p53, which regulate cell growth, circadian rhythm, inflammation and cell cycle, respectively [35,36,37] (Figure 2).

The interplay between NAMPT and SIRT1 is very intricate and well-regulated that allows the control of several cellular events and physiologic processes, such as the circadian rhythm. Visfatin is required in the regulation of the circadian gene expression [39]. This process involves SIRT1, which modifies NAMPT expression by affecting NAMPT promoter to maintain the availability of its own cofactor NAD^+^ [39]. The relationship between visfatin and SIRT1 might provide an insight into how cellular metabolism affects physiologic processes such as the circadian rhythm and even more complex processes such as senescence.

##### PARPs/MARTs

PARPs are a family of enzymes involved in the post-translation modification of target proteins by introducing ADP-ribose (ADPr) moiety [40]. They can exist as poly (ADP-ribose) polymerase (PARPs) or mono (ADP-ribose) transferase (MARTs) depending on whether they produce a poly(ADP-ribose) (PAR) or a mono(ADP-ribose) (MAR) [41]. PARPs functional roles include cell division, transcriptional and post-translational regulation [40,42,43]. In addition to regulating cell death and survival, PARPs have been implicated in cellular responses to environmental and metabolic insults (oxidation), such as DNA repair and heat shock proteins [40,42,43,44]. Moreover, PARPs were found to be critical regulators for eukaryotic physiology [45]. PARPs plays a critical role in maintenance of cell proliferation, DNA integrity, proper gene expression, cell motility and are therefore essential for cell viability [44,45].

Given that PARPs utilize NAD+ as substrates to catalyze their reactions [40,41,42], it is not unlikely to conclude that visfatin is a key player in PARPs effects and that any dysregulation in visfatin might affect NAD levels and thereby influence PARPs regulatory role (Figure 2). Mechanistically, it has been shown that the decrease in NAD+ levels following NAMPT inhibition is modulated by PARPs rather than SIRT 1 [46]. Additionally, visfatin has been found to maintain cell viability through PARP1 activation [47]. PARPs, in particular PARP alpha, has been reported to be an important regulator of visfatin’s expression in hepatic tissue, suggesting an intricate relationship between PARPs and visfatin [48].

On the other hand, inhibition of NAMPT downregulates many proteins including antioxidants, catalases, and most importantly PARP 1 [49], resulting in decreased cell survival and mitigated the cellular responses to stress. Therefore, inhibition of visfatin increases susceptibility to oxidative insults and disrupts cellular growth [49]. Following its enzymatic role in NAD^+^ biosynthesis, the inhibition of visfatin will indeed affect NAD levels and bioenergetics.

##### CD38 and CD157

**Figure 3:**
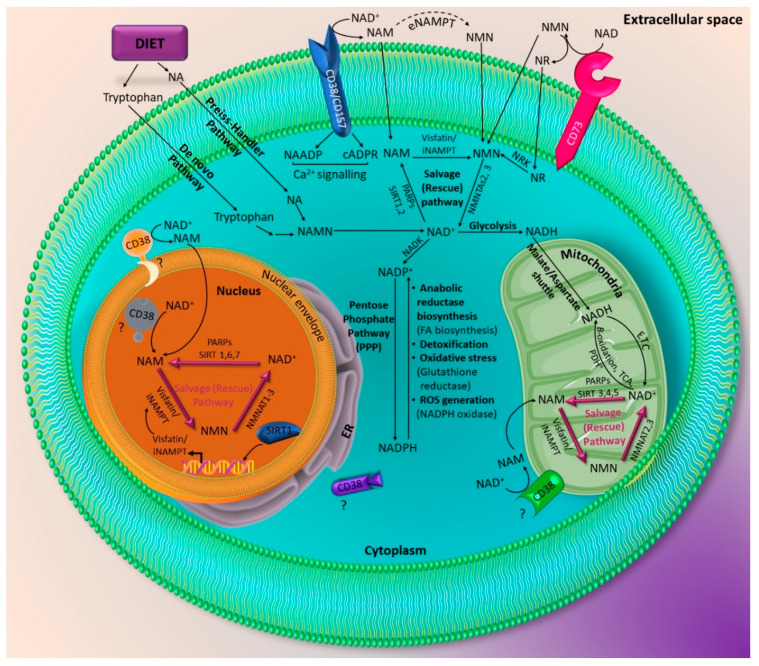
Various physiological roles of intracellular visfatin (iNAMPT). iNAMPT can be located in the cytoplasm, nucleus, and mitochondria. iNAMPT triggers its effects by regulating the levels of the core molecule NAD^+^. NAD^+^ levels are maintained through the de novo, Preiss–Handler, and salvage pathways. NAD^+^ is heavily converted to NAM by several cellular NAD^+^ dependent enzymes which include SIRT 1, 2 in the cytoplasm, SIRT 1, 6, 7 in the nucleus, and SIRT 3, 4, 5, in the mitochondria and PARPs. NAM will be converted to NMN via iNAMPT which is the rate limiting step in the salvage pathway. NAD^+^ can be regenerated from NMN via nicotinamide mononucleotide adenylyltransferases (NMNATs). The ectoenzyme CD38 converts NAD^+^ to NAM to produce NAADP and cADPR involved in intracellular Ca^2+^ signaling. CD38 can be also present on inner/outer mitochondrial membrane or inner/outer nuclear envelope. NAD^+^ can be metabolized extracellularly to NMN via the ectoenzyme CD73. NAD^+^ is a core molecule that plays a role in basic cellular metabolism. NAD^+^ is a key substrate for glycolysis, the tricarboxylic acid cycle (TCA), pyruvate dehydrogenase (PDH), beta-oxidation yielding NADH. The reduced form NADH can be regenerated via electron transport chain (ETC). NAD^+^ exist also in a phosphorylated form NADP+. The reduced form NADPH determines the anti-oxidative power of the cell. It is involved in anabolic biosynthesis (fatty acid (FA) biosynthesis), detoxification, cellular responses during oxidative stress via glutathione reductase, and protection against reactive oxygen species (ROS) via NADPH oxidase. NR: nicotinamide riboside.

CD38 is a membrane bound protein with multi-enzymatic functions [50,51,52]. It is ubiquitously expressed in different mammalian tissues [53,54]. Its major enzymatic function is hydrolysis of NAD making it a major mammalian NADase [55]. Its catalytic functions have been implicated in the metabolism of two distinct Ca^2+^ mobilization messengers: cyclic ADP-ribose (cADPR) and nicotinic acid adenine dinucleotide phosphate (NAADP) [55,56,57,58,59,60,61]. CD38 may regulate major metabolic and cellular processes by regulating NAD^+^ levels [55]. It is present in the plasma membrane of cells and may be present intracellularly in mitochondrial membrane [62]. Additionally, it has been reported to be located in the inner membrane [63,64] and outer membrane [65] of the nuclear envelope (Figure 3). In line with these studies, other studies reported high levels of CD38 to be located in plasma membrane [66], intracellularly in the nuclear membrane and endoplasmic reticulum [67] and inside cell nuclei [66]. More importantly, CD38 has been found to be constitutively expressed in hematopoietic cells, particularly in the nucleus, and researchers suggested a regulatory role in maintaining nuclear Ca^2+^ and NAD^+^ levels [68].

NAMPT and CD38 exert opposite effects on NAD^+^ availability. In fact, NAMPT inhibition has been found to have similar metabolic consequences of CD38 expression [69]. For example, age-related decreases in NAD^+^ levels is associated with increased CD38 levels [70,71]. Thus, CD38 inhibitors and NAMPT activators constitute a promising area to maintain high levels of NAD^+^ with aging.

CD157 (BST-1), known as bone marrow stromal cell antigen-1, is another surface antigen known to possess both ADP ribosyl cyclase and cADPR hydrolase enzymatic activities similar to CD38 [72]. CD38 and its homologue CD157 are considered to be two main mammalian NADases [70]. Their ability to regulate NAD^+^ levels suggests a possible relationship with visfatin that need to be uncovered more in future studies.

The fact that visfatin is a major regulator of NAD^+^ biosynthesis makes any protein or enzyme that is dependent on NAD^+^ or NADPH, vulnerable to dysregulation by visfatin. This is not limited to sirtuin and PARPs, but also might include proteins such as catalases, anti-oxidants, DNA repair proteins and metabolic enzymes, known to be dependent on NAD^+^ and NADPH availability. Visfatin’s regulatory roles extend beyond the boundaries of the cell, eith an extracellular form of visfatin existing.

### 3.2. Extracellular Visfatin (eNAMPT)

Though its physiological role tends to be unclear, the extracellular form of visfatin, eNAMPT, has been reported to act as a cytokine. PBEF, and as an insulomimetic adipokine (visfatin) pro-inflammatory mediator, and active enzymes in addition to many other functions [5,15,16,21,73]. The fact that eNAMPT lacks a signal sequence for secretion prompted researchers to entertain the possibility that eNAMPT may be the intracellular form of visfatin released due to cell lysis or after cell death [13,74,75]. This might be able to explain its ubiquitous presence in several diseases. In the first place, it was controversial whether eNAMPT is secreted or if it is just a result of cell lysis and cell death. Then, it was found that human adipocytes produce and positively secrete eNAMPT through a nonclassical pathway [16]. Accordingly, researchers suggested that the presence of eNAMPT is not indicative of cell lysis [16]. Therefore, eNAMPT is apparently different from the intracellular form, iNAMPT. Thus, the underlying physiological role of eNAMPT remains to be elucidated. The functional roles of eNAMPT are summarized in Table 2 and discussed thereafter.

#### 3.2.1. eNAMPT/Visfatin Acting as PBEF

PBEF was the first form of visfatin to be discovered in 1994 [11]. PBEF is induced by pokeweed mitogen (PWM) (mitogen derived from *Phytolacca americana*) and more significantly by cycloheximide [11]. PBEF has ubiquitous expression in bone marrow, liver, muscles, kidney and many other cells [11]. Additionally, it enhances murine pre-B-cell colony formation by working synergistically to increase the effects of stem cell factor (SCF) and IL-7 [11]. Initially, at the moment of discovery, PBEF was isolated in phytohemagglutinin (PHA)- and PWM-activated peripheral human lymphocytes [11]. Assumed to be a soluble factor involved in B-cell development, it has been given the name pre-B-cell colony enhancing factor (PBEF) [11]. Its expression is upregulated upon activation of several immune cells including T-cells [13], monocytes [76], neutrophils [77], and macrophages [78]. This suggests a possible immunological functional role of PBEF as a secreted cytokine.

#### 3.2.2. eNAMPT/Visfatin Acting as a Cytokine

Visfatin originally, when discovered as PBEF, was believed to be an immune modulating cytokine [11]. It has been reported to regulate about 50 different inflammatory genes in peripheral blood mononuclear cells (PBMCs) [21]. In line with this, visfatin has been proven to stimulate the release of many inflammatory mediators [4,71]. Additionally, it has been shown to induce monocyte chemoattractant protein 1 (MCP-1) production [79] and matrix metalloproteinases (MMPs) expression [80]. Visfatin is implicated in the activation of many inflammatory pathways such as NF-κB [4], mitogen-activated protein kinase (MAPK) and phosphatidylinositol 3 kinase (PI3) [81]. Visfatin also may act as a cytokine mediating vascular remodeling by upregulating vascular endothelial growth factor (VEGF) [82] and fibroblast growth factor 2 (FGF-2) [83]. Visfatin-induced cytokine production in leukocytes has been also linked to p38 mitogen-activated protein kinase (p38MAPK) and NF-κB p65 signaling pathways [71]. Therefore, it is clear that visfatin has abundant wide ranging functions beyond immune modulation.

#### 3.2.3. eNAMPT/Visfatin Acting as Insulinomimetic Adipokine

In 2005, a study discovered that PBEF/NAMPT is secreted by visceral fat and hence, has been denoted visfatin [15]. It has been shown that visfatin/eNAMPT elicited insulomimetic effects via binding to and activating insulin receptor in hepatocytes, myocytes and adipocytes [15]. Similar to insulin, visfatin/eNAMPT exerted a glucose-lowering effect and enhanced glucose transport and lipogenesis [15]. Moreover, it increased insulin sensitivity in diabetic mice [15]. Additionally, it has been shown that elevated blood glucose levels resulted in increased plasma PBEF/visfatin, which was abrogated by co-infusion of insulin or somatostatin [84]. However, with the retraction of some of these data [85], the involvement of insulin receptors in mediating visfatin/eNAMPT’s actions became controversial. In this line, a subsequent study revealed that visfatin/eNAMPT-induced increase in skeletal muscle glucose transport does not involve the classical insulin signaling pathways [86].

## 4. Relevance of Visfatin to Metabolic Diseases

Many studies emerged suggesting possible associations between visfatin and metabolic disorders. In fact, one meta-analysis regarded visfatin as a promising biomarker for several metabolic disorders including diabetes, insulin resistance, and obesity [87].

### 4.1. Visfatin and Diabetes

One of the first metabolic disorders to be linked to visfatin is diabetes. Several studies have reported association between visfatin levels and various types of diabetes ranging from gestational [88], type 1 [89,90], and type 2 diabetes [89,91,92,93,94]. Another investigation reported increased circulating visfatin with progressive B-cell deterioration [89]. In contrast, many other studies had opposite findings and reported no association between visfatin and diabetes. In this regard, low circulating visfatin levels were found in gestational [95] and other forms of diabetes [96]. Additionally, one study reported no significant difference between circulating visfatin levels in type 2 diabetic patients compared to matched healthy individuals [97]. Moreover, no association between circulating visfatin and insulin sensitivity or glucose tolerance has been found in other studies [94,98,99,100,101].

Visfatin may play a role in the pathogenesis of diabetes through interaction with the insulin receptor. Indeed, through binding to insulin receptor, recombinant visfatin was found to phosphorylate tyrosine and insulin substrate-1 and -2, thereby enhancing glucose uptake [15]. Interestingly, visfatin was found to bind to insulin receptor with an affinity comparable to that of insulin, albeit at a different binding site [15]. However, visfatin could activate downstream signaling at a 10-fold lower molar concentration than insulin [15]. Similarly, the insulin-mimetic effects of visfatin, including increasing glucose uptake in human osteoblasts, was also demonstrated [102]. Relevantly, this visfatin-enhanced glucose uptake was also reported in SGBS pre-adipocytes [71]. However, increasing visfatin concentration from 100 ng/mL to 2 µg/mL did not further enhance glucose uptake in pre-adipocytes [71]. Collectively, this suggests a possible compensatory role for visfatin in diet or obesity-induced diabetes.

### 4.2. Visfatin and Obesity

A similar profile of contradictory results has been documented with regards to analyses correlating visfatin and obesity. Whereas some studies have reported positive correlations between visfatin and obesity [93,95,96], others have demonstrated low plasma visfatin levels in patients with obesity [99,100]. However, one reading described visfatin to be associated with type 2 diabetes rather than obesity [97]. In contrast, visfatin levels were comparable in obese nondiabetics and lean controls, but were significantly upregulated in obese type 2 diabetic patients, suggesting that visfatin is related to type 2 diabetes, rather than to obesity. On the other hand, no association between circulating visfatin levels and metabolic disorders, such as diabetes, various types of obesity (generalized, or abdominal and subcutaneous, or visceral), or even dyslipidemia has been documented [96].

Despite the contradictory data available regarding visfatin and obesity, some studies reported possible roles of visfatin in obesity-associated injury. Inflammasome activation was shown to be a central player in the pathogenesis of adipose tissue inflammation, insulin resistance (IR), and obesity-associated metabolic diseases [103]. More importantly, inflammasome activation was shown in many instances to be adipokine-driven [103]. Furthermore, the ability of visfatin to mediate obesity-induced podocyte injury via NOD-, LRR- and pyrin domain-containing protein 3 (NLRP3)-inflammasome activation has also been shown [104]. Additionally, visfatin was shown to mediate arterial inflammation and endothelial dysfunction during early stages of obesity, via an NLRP3 inflammasome dependent endothelial inflammatory response [105]. Similarly, visfatin-induced vascular dysfunction in mice was shown to involve NLRP3-inflammosome and paracrine IL-1ß via a NAMPT-dependent Toll-like receptor 4 (TLR4)-mediated pathway [106]. Another study found that visfatin-induced endothelial NLRP3-inflammasomes may result in the production of high mobility group box protein 1 (HMGB1) [107]. Consequently, HMGB1 can disrupt inter-endothelial junctions and increase paracellular permeability of the endothelium via paracrine and autocrine signaling, resulting in early stage endothelial injury during metabolic disorders such as obesity [107]. Together, these findings suggest that the NLRP3 inflammasome, HMGB1, TLR4, and possibly some other mediators might serve as promising therapeutic targets to counter visfatin-mediated vascular injury associated to obesity.

A very recent paper reported a potentially important new role for visfatin in the context of metabolic disease. The report shows that visfatin upregulates extracellular matrix (ECM) proteins including osteopontin, collagen type VI, MMP-2, and MMP-9 in pre-adipocytes [108]. Given the documented role of ECM protein in tissue fibrosis, the authors suggested adipose tissue fibrosis as a possible link between visfatin and obesity-associated fibrosis and insulin resistance [108].

### 4.3. Visfatin and PCOS

Polycystic ovary syndrome (PCOS), a common endocrine metabolic disorder in women, characterized by hyperandrogenism, obesity, impaired lipid metabolism and insulin resistance [109]. Several studies demonstrate higher visfatin/NAMPT plasma concentrations in PCOS women than those in matched controls [109,110,111,112], suggesting its implication in the pathogenesis of PCOS. In this context, visfatin/NAMPT plasma concentrations have been shown to be positively correlated with glucose level, insulin and insulin resistance [109], body mass index and the log free androgen index [113], as well as many lipid profile parameters, including total cholesterol, LDL cholesterol, triglycerides, lipoprotein(a) [114]. Moreover, serum eNAMPT were found to be strongly correlated with free testosterone levels suggesting a possible role of visfatin in the pathogenesis of PCOS [115]. Moreover, visfatin was found to be an independent predictor of fibromuscular dysplasia (FMD) in patients with PCOS [115].

A role for visfatin in the pathogenesis of endothelial dysfunction in PCOS has been suggested. This was thought to be due to increased inflammation associated with visfatin. Indeed, studies reported a correlation between visfatin levels and pro-inflammatory markers. For instance, serum visfatin levels were significantly associated with C-reactive protein (CRP) and white blood cell (WBC) levels; however, no association was found with PCOS [116]. This prompted the authors to propose that visfatin acts as a possible pro-inflammatory cytokine in women with PCOS, which may then explain how visfatin contributes to endothelial dysfunction in PCOS [116]. Mechanistically, visfatin may mediate endothelial dysfunction in PCOS by inducing the expression of pro-angiogenic factors such as VEGF and MMP-9 [117]. Nonetheless, much remains to be investigated about the interplay between visfatin, inflammation, endothelial dysfunction and PCOS.

Because a relationship between visfatin and insulin resistance had been established, it was tempting to assume such a relationship exists in women with metabolic diseases. Interestingly, higher plasma visfatin levels were reported in PCOS women with insulin resistance [112]. These elevated levels in PCOS patients compared to control women of similar age and body mass index (BMI) may suggest a possible role for visfatin in mediating insulin resistance in PCOS [112]. While it would be tempting to propose that visfatin might play a role in the pathogenesis of PCOS by mediating hyperandrogenism, obesity, and insulin resistance, one cannot overlook findings in other studies where no causative correlation between visfatin levels and PCOS can be conclusively presumed [116].

### 4.4. Visfatin in Clinical Studies

The variations observed in plasma visfatin levels in several metabolic disorders suggests a possible role in the pathogenesis of these disorders and therefore have therapeutic implications. Many research teams have indeed started to investigate visfatin levels in their clinical trials. Several clinical studies targeting metabolic disorders started to include visfatin in their criteria for evaluating therapeutic efficacy.

One randomized clinical trial studied the effects of metformin immediate release compared with metformin extended release on glycemic control in type 2 diabetes mellitus (T2DM) [118]. The authors observed increased levels of visfatin in patients randomized to metformin extended release [118]. Another trial showed a reduction in visfatin serum levels after metformin administration in PCOS women [119]. In contrast to these studies, one trial reported no variation in serum visfatin levels despite improved glycemic control in response to slow-release and regular-form metformin in T2DM [120]. Similarly, one clinical study detected no significant changes in visfatin levels when rosiglitazone or metformin monotherapy was utilized in T2DM patients [121]. Similarly, no change in visfatin serum levels was noted when PCOS women were treated with pioglitazone [122]. Additionally, no change in visfatin plasma levels was detected in response to pioglitazone or metformin treatment despite improvement in insulin sensitivity and glycemic regulation in naïve T2DM (newly diagnosed and untreated T2DM) [123].

Several human studies are now paying closer attention to visfatin levels when metabolic diseases are investigated. For instance, several studies investigating the effect of L-carnitine supplementation on glucose oxidation and insulin resistance markers in T2DM have considered visfatin levels as an important parameter. Indeed, l-carnitine was found to reduce levels of the adipokine visfatin in many trials when combined with a T2DM regimen. In one study, addition of L-carnitine to glimepiride was found to reduce visfatin levels in T2DM patients [124]. Similar results were achieved in obese diabetic patients when treated with orlistat and L-carnitine [125] and in diabetic patients when treated with sibutramine and l-carnitine [126].

These studies further support the relevance of visfatin in these diseases, and warrant further investigations that may present this adipokine as an attractive target in the fight against cariovasculometabolic disease. Visfatin might serve as a biomarker for lipid profile control in metabolic disorders. Its plasma levels may be used to track the therapeutic progress in patients with metabolic diseases. The possibility of visfatin to play a role as a prognostic factor also needs to be investigated.

## 5. Conclusions

Visfatin is a novel adipokine which is abundantly expressed in visceral fats. It elicits insulomimetic actions, and consequently its plasma level is closely associated with many metabolic diseases including obesity, diabetes mellitus and PCOS. Given that many of the metabolic diseases are major risk factors and contributors to increased morbidity and mortality from cardiovascular disease (CVD) [127,128], further investigation of visfatin with regards to its implication and therapeutic target potentials in cardiovascular–metabolic disorders is warranted. Obviously, the role of serum visfatin in metabolic diseases remains debatable [129]. Nevertheless, controversial studies do not rule out the possibility of an association between visfatin and these metabolic disorders, but rather suggest the existence of specific metabolic conditions that dictate the plasma concentration of visfatin. In fact, the ubiquitous expression of visfatin in many cells and tissues makes it complex and difficult to make any association using its plasma concentration. There might be other players controlling the visfatin plasma concentration and concealing any possible role. Moreover, the existence of some limitations in different immunoassays used for the detection of visfatin serum levels may contribute to the observed discrepancies [130]. Three immunoassays comprised of an enzyme immunoassay (EIA), radioimmunoassay (RIA), and enzyme linked immunosorbent assay (ELISA) were used in a study to detect visfatin [130]. A significant disparity in visfatin concentration has been found between EIA and RIA and between EIA and ELISA. Each of the immunoassays has its own limitations with the ELISA being the most sensitive but with a narrow detection range [130]. Therefore, the development of sensitive immunoassays with wider detection ranges to detect serum visfatin accurately may be necessary to explain those controversial observations and unwind any possible correlation. Additionally, plasma visfatin levels are not necessarily true representatives of the tissue activity.

## Figures and Tables

**Table 1:** Functional role of intracellular visfatin (iNAMPT).

NAD^+^-Dependent Cellular Signaling	Functional Outcomes
Sirtuins	Cell division/proliferation Cell differentiation Cell survival/apoptosis Life span Inflammation Cell metabolism/expenditure
CD38/CD157	Ca^2+^ signaling
PARPs/MARTs	Cell proliferation DNA repair/DNA integrity Cellular responses to environmental/oxidative stress Proper gene expression Mitochondrial health Transcriptional and post-translational regulation

**Table 2:** Functional roles of extracellular visfatin (eNAMPT).

Functional Role	Possible Outcomes
PBEF	Enhances murine pre-B-cell colony formation Upregulates SCF and IL-7
Cytokine	Inflammatory pathways: NF-κB, MAPK, PI3 Vascular remodeling: ↑ MCP-1, ↑ MMP, ↑ VGEF, ↑ FGF-2
Insulin-mimetic	Binds insulin receptor Increases insulin sensitivity and glucose lowering effects Enhances glucose uptake/transport Lipogenesis
